# A case report of ectopic pregnancy arising in a unicornuate uterus, accompanied by the undescended tube and ovary with double inferior vena cava

**DOI:** 10.1097/MD.0000000000021105

**Published:** 2020-07-10

**Authors:** Y.S. Jang, Y.S. Kim

**Affiliations:** Department of Obstetrics and Gynecology, Soonchunhyang University College of Medicine, Soonchunhyang University Cheonan Hospital, Cheonan, Korea.

**Keywords:** double inferior vena cava, ectopic pregnancy, fallopian tube, undescended ectopic ovary, unicornuate uterus

## Abstract

**Rationale:**

The incidence of a unicornuate uterus is 0.2% to 0.3% of the whole population. A unicornuate uterus is closely associated with obstetrical complications such as early miscarriages, ectopic pregnancy, and malpresentation.

**Patient concerns:**

A 32-year-old patient developed a rare ectopic pregnancy arising at a distal, fimbriated end of the undescended fallopian tube.

**Diagnoses:**

A transvaginal ultrasound scan revealed hemoperitoneum and no gestational sac in the uterine endometrium. A laparoscopic finding showed that high up in the right abdomen, just below the liver, an ectopic mass could be seen arising at a distal, fimbriated end of the fallopian tube, which was developed adjacent to the undescended right ectopic ovary.

**Interventions:**

After laparoscopic removal of the right salpinx, we removed it with a bag.

**Outcomes:**

One day after the operation, she was discharged without problems. Postoperative hysterosalpingography showed the unicornuate uterus with patent left and some right salpinx. Magnetic resonance imaging revealed a unicornuate uterus, right ovary at the right inferior hepatic area, a bilateral normal kidney, and double inferior vena cava.

**Lessons:**

This is the first reported case of its type. It demonstrated that ectopic pregnancy may occur in the upper abdomen, not in the pelvic cavity, in uterine anomaly, and double inferior vena cava; hence, we must thoroughly check the whole abdominal cavity. Additional imaging tests are needed after treatment to see if there are any abnormalities.

## 1 Introduction

A unicornuate uterus is caused by a failure of one Müllerian duct to develop or to migrate to its proper location. It is linked to an increase in obstetrical complications such as early miscarriages, ectopic pregnancy, abnormal fetal presentation, intrauterine growth restriction, and preterm labor.^[1]^ Despite the well-known association of ectopic ovaries and unicornuate uterus, ectopic ovaries are reported only sporadically, suggesting that many cases may go unrecognized.^[2]^ The embryologic mechanism underlying undescended ovaries is uncertain, but it might be caused by a lack of caudal descent of the gonads into the true pelvis or by a retarded differential growth of that portion of the urogenital ridge that gives rise to both the gonad and the fallopian tube.^[3]^ Although the incidence of a unicornuate uterus is very low, a correct diagnosis is mandatory, not only to be aware of the existence of an ectopic ovary and salpinx, but also to consider the reproductive performance when uterine malformations are involved.^[4]^ We introduce the first case of an ectopic pregnancy arising at a distal, fimbriated end of the fallopian tube, which developed adjacent to the undescended ectopic ovary at the inferior hepatic portion in a unicornuate uterus, and was accompanied by a double inferior vena cava.

## 2 Case report

A 32-year-old Asian female, gravida 1, para 0, was admitted to the emergency room of our clinic because of right lower quadrant abdominal pain. Her last menstrual period had been 9 weeks ago. Four weeks ago, she was received dilatation and curettage at a local clinic because there was no gestational sac in her uterus and she had slowly increasing beta-human chorionic gonadotropin (β-hCG). After dilatation and curettage, her β-hCG continuously increased from 2400 to 2800 mIU/mL, she received injected methotrexate 50 mg intramuscular. One week after the methotrexate injection, her β-hCG was decreased to 1700 mIU/mL. But, 1 week later, she had severe abdominal pain, she was admitted to the emergency room of our hospital. Her vital signs were stable and her β-hCG was 570 mIU/mL. Ultrasound examination revealed fluid collection in the anterior cul-de-sac and posterior cul-de-sac, which suggested hemoperitoneum. The endometrial thickness was 5 mm, and there was no gestational sac. The left ovary was normal, but the right ovary was not seen.

**Figure 1:**
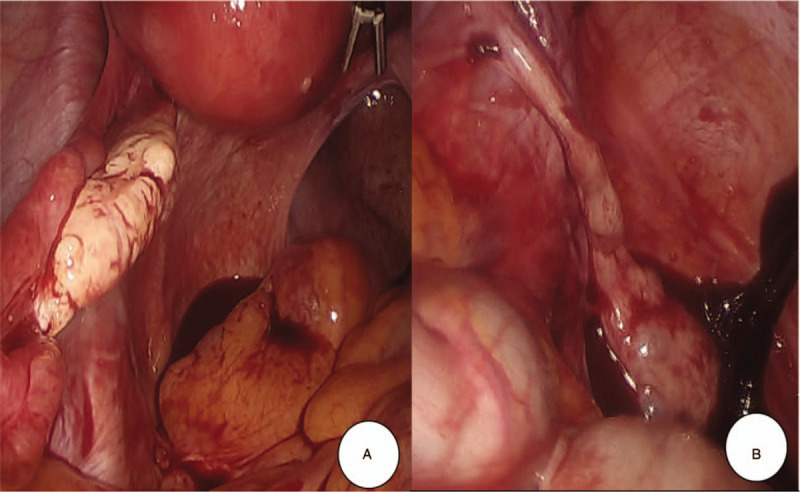
Laparoscopic findings of pelvic cavity. (A) About 500 mL of fresh blood and clots in the abdominal cavity was aspirated. Left-side salpinx and ovary were intact. No ectopic mass in pelvic cavity. (B) Some right-side salpinx was seen in the lower portion, but not the right-side fimbriae and ovary.

**Figure 2:**
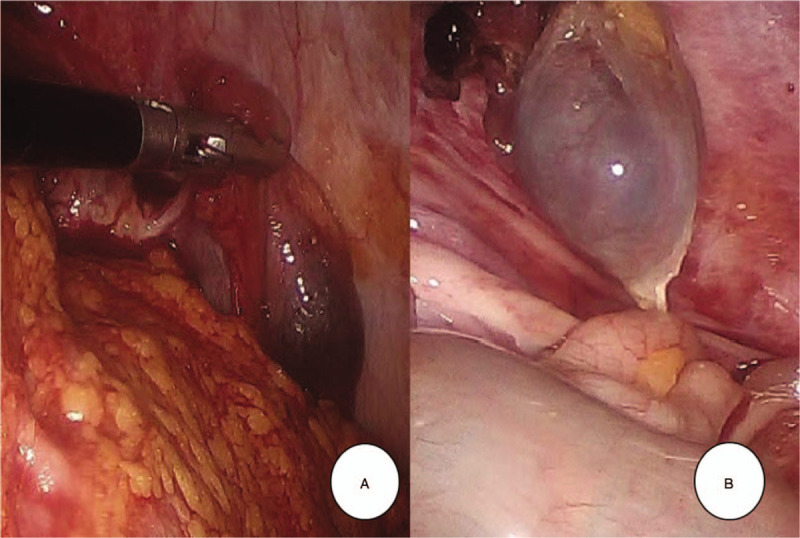
Laparoscopic findings of right upper abdomen. (A) High up in the right abdomen, just below the liver, an ectopic mass could be seen adjacent to the undescended right ectopic ovary. (B) Ectopic mass arose at a distal, fimbriated end of the fallopian tube.

At laparoscopy, about 500 mL of fresh blood and clots in the abdominal cavity was aspirated. The left side salpinx and ovary were intact (Fig. 1A). Some right-side salpinx was seen, but no right-side fimbriae and ovary in the lower portion (Fig. 1B). There was no ectopic mass in the pelvic cavity. High up in the right abdomen, just below the liver, an ectopic mass could be seen adjacent to the undescended right ectopic ovary (Fig. 2A), which arose at a distal, fimbriated end of the fallopian tube (Fig. 2B). After right salpingectomy, her β-hCG decreased from 570 to 386 mIU/mL. She discharged at postoperative 1 day without complications.

**Figure 3:**
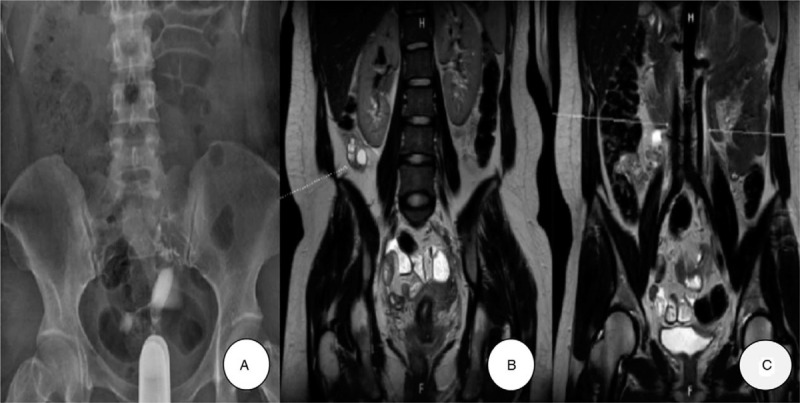
Postoperative hysterosalpingography (HSG) and magnetic resonance imaging (MRI) findings. (A) HSG showed a unicornuate uterus, preserved patency of left salpinx in the upper pole and some right salpinx in the lower pole. (B) MRI showed the right ovary at the right inferior hepatic portion (arrow). (C) MRI showed bilateral normal kidneys, and a double inferior vena cava (arrow).

After several days, the ectopic pregnancy was histologically confirmed. A postoperative hysterosalpingography showed a unicornuate uterus and preserved patency of left salpinx and some right salpinx (Fig. 3A). A magnetic resonance imaging showed the right ovary at the right inferior hepatic portion (Fig. 3B, arrow), bilateral normal kidney, and double inferior vena cava (Fig. 3C, arrow).

## 3 Discussion

Congenital malformations of the Müllerian system are probably caused by multifactorial polygenic and familial factors. Uterine malformations are the most frequent anomalies of the Müllerian ducts. They occur in 2% to 4% of fertile women.^[5]^ The prevalence of a unicornuate uterus is rather low. However, of all Müllerain defects, a unicornuate uterus is found in 3% to 13% of women. The uterus is formed during embryogenesis the fusion of the 2 Müllerian system. If one of the ducts does not develop, only 1 Müllerian duct contributes to the uterine formation. This uterus may or may not be connected to Müllerian structure on the opposite site if the Müllerian duct on that site undergoes some development. Associated defects may affect the renal system, and less common, the skeleton. Disruption of the developing local mesoderm and its contiguous somites accounts for some associated axial skeletal abnormalities like cervical skeletal fusion. It is mainly associated with renal agenesis, ureteric agenesis, ectopic ovaries, and undescended tubes.^[6]^ In this case, she had no renal agenesis but had a double inferior vena cava (IVC). *Double IVC* is a congenital variation caused by an unusual embryological development of the IVC. Usually, it is detected incidentally, while evaluating genitourinary anomalies. IVC duplication associated with Müllerian anomalies is an extremely rare condition, mainly with unilateral renal agenesis.^[7]^ There are no reports of a unicornuate uterus with bilateral normal kidneys in a double IVC patients such as this. That is the rarity of this case. A unicornuate uterus forms when the development of a paramesonephric duct is impaired. There are 2 classification systems widely used. The one is Buttram and Gibbons, and the other is European Society of Human Reproduction and Embryology-European Society for Gynaecological Endoscopy.^[8]^ A unicornuate uterus is a type II classification with unilateral hypoplasia or agenesis, which can be further subclassified into communicating, no cavity, and no horn.^[9]^ However, undescended ovaries and fallopian tubes are not mentioned in either classification, which is the most unfortunate drawback in the above classification. Ectopic or undescended ovaries are characterized by the attachment of the upper pole to an area above the level of the common iliac vessels, and they are very rare congenital defects.^[10]^ But, the incidence is reported to be more than 40% in cases with a unicornuate uterus.^[11]^ Ectopic pregnancies in such misplaced organs can occur, are very difficult to diagnose, and are associated with important maternal morbidity and mortality.^[12]^ The embryologic mechanism underlying undescended ovaries is uncertain, but may be caused by a lack of caudal descent of the gonads into the true pelvis or by a retarded differential growth of the portion of the urogenital ridge that gives rise to both the gonads and the fallopian tubes. During the 3rd month of fetal life, the developing ovaries descend from a position near the kidneys to their final position in the pelvis. This descent is guided by the gubernaculum, which is attached to the uterus, forming the utero-ovarian and round ligament. When a proper attachment of the gubernaculum is not possible, that leads to failed descent of that ovary. The fallopian tube is frequently not attached to the uterus in these cases, and only the distal, fimbriated end of the tube develops adjacent to the undescended ovary. Our case was similar. Several cases reveal the phenomenon of transperitoneal gamete/embryo migration.^[13]^ The absence of a tubal connection with the unicornuate uterus and the presence of a corpus luteum in the contralateral ovary may serve as evidence for it. It remains unknown whether chemotactic or other factors are involved in the transport of gametes or embryos across the peritoneal cavity to reach the contralateral and heterotopic tube.^[14]^ It seems quite mysterious. In summary, an ectopic pregnancy in an undescended fallopian tube is a rare condition but can be found frequently with a unicornuate uterus. It can be evidence of transperitoneal gametes or embryo transmigration. Such cases are very difficult to diagnose. So, clinicians should carry out ultrasound examination of the whole-abdomen carefully. A unicornuate uterus is a Müllerian anomaly with prognostic implications for poorer outcomes during pregnancy. Although it is unclear whether interventions before conception or early in pregnancy, such as resection of the rudimentary horn or prophylactic cervical cerclage, decidedly improve obstetrical outcomes, current practice suggests that such interventions may be helpful. Women presenting with a history of this anomaly should be considered high-risk obstetrical patients.^[15]^ Research is needed to find out whether artificial reproductive treatment can improve pregnancy outcomes in women with a unicornuate uterus.^[16]^ Currently our patient is also trying hard to get pregnant.

## Acknowledgment

The authors are grateful to Soonchunhyang University Cheonan Hospital for its assistance and encouragement.

## Author contributions

Conceptualization, Data curation, Investigation, Writing the original draft preparation: Kim YS. Writing review: Jang YS.

All authors read and approved the final manuscript.

**Writing – review & editing:** Y.S. Jang.
